# The impact of the SARS-CoV-2 pandemic on referral characteristics in a national tertiary spinal injuries unit

**DOI:** 10.1007/s11845-021-02678-0

**Published:** 2021-06-11

**Authors:** Louis O’Halloran, Daniel P. Ahern, Jake M. McDonnell, Gráinne M. Cunniffe, Michael K. Dodds, Frank Lyons, Noelle Cassidy, Marcus Timlin, Seamus Morris, Keith Synnott, Joseph S. Butler

**Affiliations:** 1 National Spinal Injuries Unit, Mater Misericordiae University Hospital, Eccles Street, Dublin 7, Ireland; 2 School of Medicine, University College Dublin, Belfield, Dublin, Ireland; 3 School of Medicine, Trinity College Dublin, College Green, Dublin, Ireland; 4 School of Medicine, Royal College of Surgeons in Ireland, 123 St Stephen’s, Green, Dublin, Ireland

**Keywords:** Coronavirus, COVID-19, Referrals, SARS-CoV-2, Spinal injury, Spine surgery

## Abstract

**Background:**

The SARS-CoV-2 pandemic has had profound implications on healthcare institutions.

**Aims:**

This study aims to assess and compare referral patterns during COVID-19 to corresponding dates for the preceding 3 years (2017–2019), in order to preemptively coordinate the logistics of the surgical unit for similar future experiences.

**Methods:**

Retrospective review for our institution, a national tertiary referral centre for spine pathology. Two distinct time-points were chosen to represent the varied levels of social restriction during the current pandemic: (i) study period 1 (SP1) from 11 November 2020 to 08 June 2020 represents a national lockdown, and (ii) study period 2 (SP2) from 09 June 2020 to 09 September 2020 indicates an easing of restrictions. Both periods were compared to corresponding dates (CP1: 11 March–08 June and CP2 09 June–09 September) for the preceding 3 years (2017–2019). Data collected included age, gender, and mechanism of injury (MOI) for descriptive analyses. MOIs were categorised into disc disease, cyclist, road-traffic-accident (RTA), falls < 2 m, falls > 2 m, malignancy, sporting injuries, and miscellaneous.

**Results:**

All MOI categories witnessed a reduction in referral numbers during SP1: disc disease (−29%), cyclist (−5%), RTAs (−66%), falls < 2 m (−39%), falls > 2 m (−17%), malignancy (−33%), sporting injuries (−100%), and miscellaneous (−58%). Four of 8 categories (RTAs, falls < 2 m, malignancy, miscellaneous) showed a trend towards return of pre-lockdown values during SP2. Two categories (disc disease, falls > 2 m) showed a further reduction (−34%, −27%) during SP2. One category (sporting injuries) portrayed a complete return to normal values during SP2 while a notable increase in cyclist-related referrals was witnessed (+ 63%) when compared with corresponding dates of previous years.

**Conclusion:**

Spinal injury continues to occur across almost all categories, albeit at considerably reduced numbers. RTAs and falls remained the most common MOI. Awareness needs to be drawn to the reduction of malignancy-related referrals to dissuade people with such symptoms from avoiding presentation to hospital over periods of social restrictions.

## Introduction

The SARS-CoV-2 pandemic, more commonly known as COVID-19, has had profound implications on global healthcare systems. Several necessary precautions have been adopted to mitigate the transmission of the virus within hospitals, including redirection of resources, reimagination of facilities as isolation units, and cancellation of elective procedures [1–3]. Additionally, reports indicate that fewer prospective patients are presenting for consultation, and an increased proportion of existing patients are failing to present for scheduled appointments, potentially a consequence of fear regarding the morbidity and mortality associated with contracting COVID-19 [2, 3].

The situation at our institution, a national tertiary referral centre for spine pathology, reflects these reports. There has been a dramatic reduction in the amount of patient contact in clinics with patients being assessed virtually where possible [4]. While the efficacy of telemedicine is evident throughout literature, it does not encompass the trauma cohort which represents a significant proportion of referrals to our institution [4–6]. Due to the neurological and vascular sequelae reported with delayed surgery in spine trauma patients, appropriate strategies must be implemented to ensure adequate orthopaedic spine care can be maintained and delivered in the trauma setting [7]. Notably, preoperative preparation of the operating theatre has changed considerably, with an increased need for personal protective equipment (PPE) and lengthy theatre decontamination protocols following procedures on COVID-19-positive or COVID-19 query patients, resulting in logistical delays [7]. Such obstacles coupled with increased demand for ventilators, anaesthetic staff, and hospital space for COVID-19 patients are greatly limiting operating capabilities and requiring constant modification to the daily logistics of the spinal unit [8].

In order to aid in the event of future COVID-19 waves or pandemics, we examine here the volume and mechanism of injury (MOI) of spinal injury patients referred to our institution over periods of varied levels of social restrictions during the pandemic.

## Methods

A retrospective study was performed at our institution, a national tertiary referral centre for spine pathology. Referral characteristics were collected and collated for patients referred to our institution during varied levels of social restrictions implemented to reduce the incidence and transmission rates of COVID-19 and compared with previous years.

Two distinct time-points were chosen to represent the varied levels of social restrictions. Study period 1 (SP1) indicates a 3-month time period from 11 March 2020 to 8 June 2020, which coincides with the first national lockdown. Study period 2 (SP2) represents a 3-month time-period from 9 June 2020 to 9 September 2020, which coincides with an easing of restrictions, to elucidate if there was an associated change in referral characteristics. SP1 and SP2 were compared with respective corresponding dates over a 3-year period. SP1 was compared to comparison period 1 (CP1), which represents referrals in 2017, 2018, and 2019 from 11 March to 8 June. SP2 was compared to comparison period 2 (CP2), which represents referrals in 2017, 2018, and 2019 from 9 June to 9 September. Together, SP1 and CP1 represent group 1 (G1), while SP2 and CP2 represent group 2 (G2). This was the preferred method of comparison as different referral characteristics and mechanisms of injury can vary throughout a calendar year, for cultural or environmental reasons. For example, the majority of sports may be conducted during the summer months, and therefore one might expect a natural increase of sporting-related spinal injuries during the summer months than compared to winter months. Thus, the authors prefer not to directly compare total referral numbers for SP1 and SP2. Instead, SP1 and SP2 may be indirectly compared in terms of an increase or reduction (%) in referral numbers with regards to their respective comparison periods (CP1 and CP2).

All referrals during aforementioned respective time-points were included for analysis. Data collected included age, gender, and mechanism of injury. SPs and CPs were compared for both descriptive analyses. All graphs were generated using GraphPad Prism software (version 8.4.1). The aim of this study is to compare referral characteristics during the COVID-19 for two time-points reflective of different levels of social restrictions to their respective corresponding dates over the three preceding years, to elucidate referral patterns during a global pandemic in order to pre-emptively coordinate the logistics of the surgical unit.

## Results

A total of 1954 patients were included in the study. The first group (group 1) includes those from study period 1 (SP1) and comparison period 1 (CP1) and amounted to 886 patients. The second group (group 2) includes those from study period 2 (SP2) and comparison period 2 (CP2) and amounted to 1068 patients.

### Group 1

**Fig. 1:**
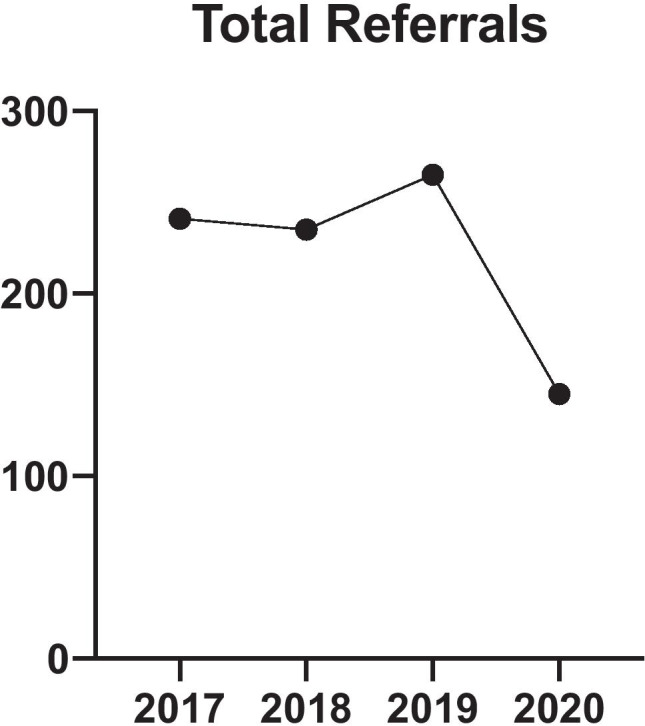
Total referrals for the 11th of March to the 8th of June for years 2017–2020

In total, there was a 41% reduction in the number of patients being referred to our institution over SP1 compared to the average from CP1. There were 145 referrals in SP1 compared to an average of 247 referrals (2017: 241, 2018: 235, 2019: 265) during CP1 (Fig. 1). The average age of referred patients over the SP1 was 57 years, compared to an average of 60 over the previous 3 years during CP1. Over study period 1, 53% of the referrals were male and 47% female, compared to an average of 57% male and 43% female over CP1.

**Fig. 2:**
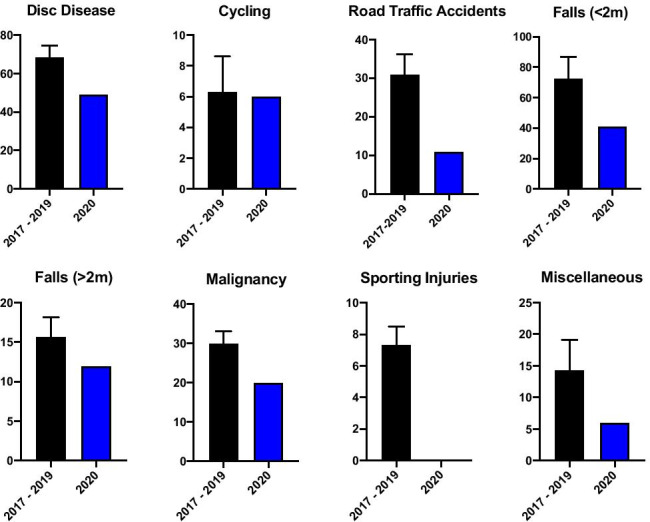
Comparative descriptive statistics for respective group 1 mechanisms of injury referral numbers in 2020 and the preceding three years 2017–2019 (average shown, with range)

**Fig. 3:**
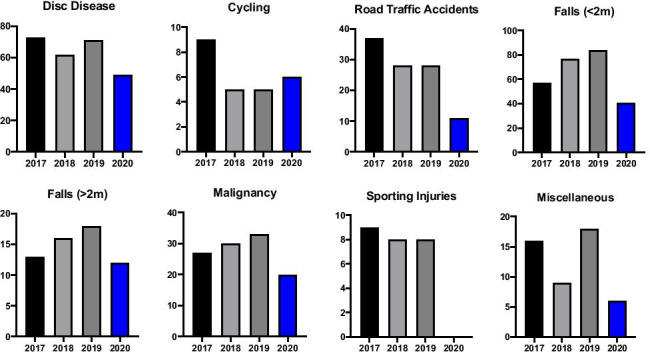
Comparative descriptive statistics for respective group 1 mechanisms of injury referral numbers in 2020 and the individual preceding 3 years (2017–2019)

We received 49 referrals during SP1 related to disc disease, amounting to a 29% decrease when compared to CP1. The number of injuries as a result of cycling did not change notably over SP1 with 6 injuries in total, in line with the CP1 average. RTA related referrals decreased significantly with only 11 cases presenting to our institution during SP1, a 66% decrease on the previous three years. The number of fall-related referrals was reduced also. Referrals for falls from a height of < 2 m decreased by 39% to 41 cases in SP1, while falls from a height of > 2 m were reduced by 17% resulting in 12 referrals during SP1 as opposed to an average of 15 in CP1. Comparative referral numbers for respective mechanisms of injury for group 1 (G1) are outlined in Figs. 2 and 3.

### Group 2

**Fig. 4:**
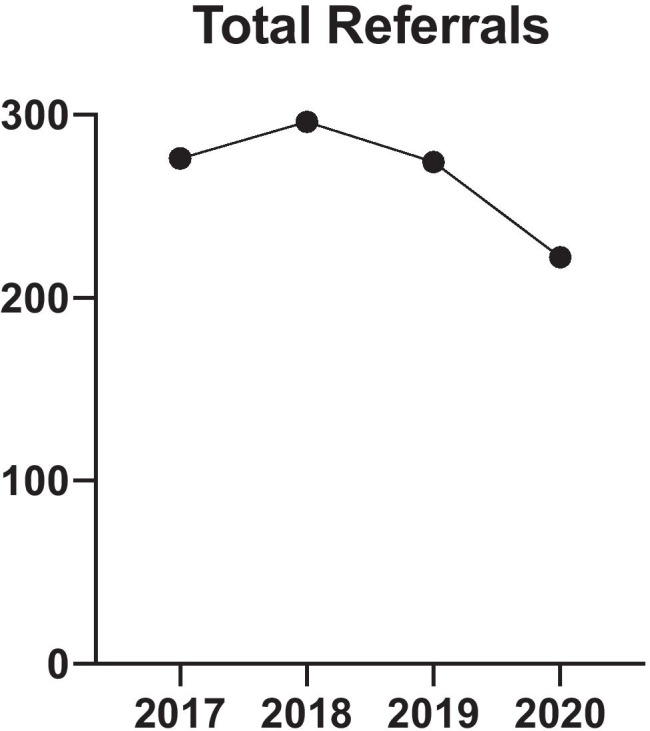
Total referrals for the 9th of June to the 9th of September for years 2017–2020

In total, there was a 21% reduction in the number of patients referred to our institution over SP2 relative to CP2. There were 222 referrals in SP2 compared to an average of 282 referrals (2017: 276, 2018: 296, 2019: 274) during CP2 (Fig. 4). The average age of patients referred for SP2 was 57 years, while the average age for CP2 was 59. Sixty-three percent were male while 37% were female for the SP2 cohort; for CP2, 57% male and 43% female.

From the total of 145 referrals in SP1, there were 20 patients presenting with referral related malignancy, accounting for 14% of referrals. From 2017 to 2019, there were 27, 30, and 33, respectively. This represents a 33% reduction in the number of patients referred with malignancy related injury in SP1 relative to CP1. There were no sporting injuries referred during SP1, compared to an average of 8 sporting-related spinal injuries referred each year. The category “miscellaneous” is comprised of spinal injuries that did not meet criteria for inclusion in any of the other MOI groupings. It contains a broad spectrum of injury largely relating to building site and farmyard accidents and also encompassing some cases of both accidental and non-accidental injury. The numbers in this group are small and so their significance is limited; however, we experienced a 58% drop in referrals in this category, with only 6 cases in 2020 relative to an average of 14 over CP1. All referrals for concerned time periods are outlined in Fig. 3.

**Fig. 5:**
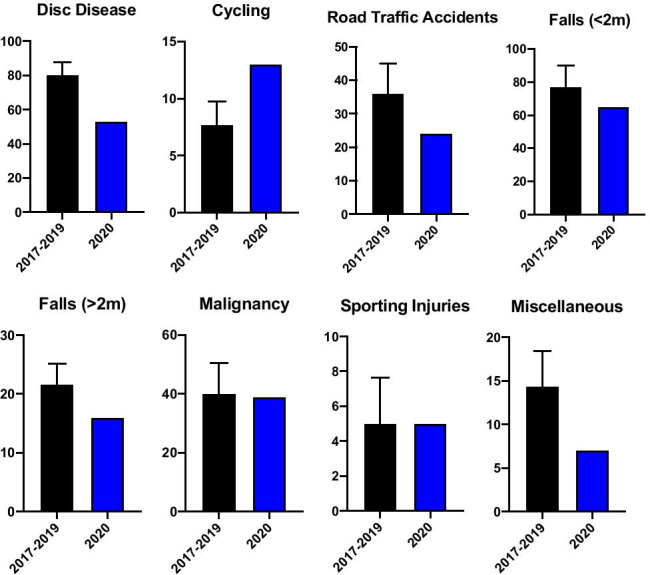
Comparative descriptive statistics for respective group 2 mechanisms of injury referral numbers in 2020 and the preceding 3 years 2017–2019 (average shown, with range)

There were 53 referrals related to disc disease during SP2, a reduction of 34% compared to the previous 3 years. While the numbers involving cyclists are relatively small, there was a notable increase in referrals of 63% (13 vs. 8) relative to the preceding 3-year average for CP2. RTA referrals remained decreased at 33% below the average with 24 patients referred over SP2. The breakdown of falls in group 2 was somewhat different to those noted in group 1. Over SP2, there were 65 falls from a height of < 2 m, a reduction of 16% during CP2. However, falls from a height > 2 m decreased from an average of 22 referrals during CP2 to 16 reported during SP2, accounting for a decrease of 27% (Fig. 5).

**Fig. 6:**
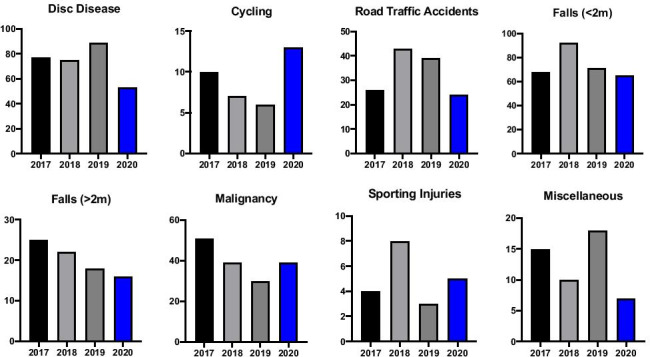
Comparative descriptive statistics for respective group 2 mechanisms of injury referral numbers in 2020 and the individual preceding 3 years (2017–2019)

The number of patients presenting with a malignancy related referral over SP2 were 39, compared to a preceding 3-year average of 40 patients. This represents a 2.5% decrease for SP2 relative to CP2. Malignancy-related referrals were on a downward trend with regards to referral numbers over the preceding three years with 51, 39, and 30 referrals reported in 2017, 2018, and 2019, respectively. Interestingly, compared to the preceding year (2019) alone, malignancy-related referrals actually increased over SP2. Sporting injuries were similar for SP2 compared to CP2, with an average of 5 referrals a piece. As was the case with group 1, the category “miscellaneous” is comprised of spinal injuries that did not meet criteria for inclusion in any of the other MOI groupings. In this category, we experienced a drop of 50% in referrals over SP2 relative to CP2, with 7 patients presenting during SP2 compared to an average of 14 during CP2. All referrals are outlined in Fig. 6.

### Study period 1 vs study period 2

For the degenerative disc-disease referral category, there were 49 referrals during SP1 (−29%) compared to 53 (−34%) during SP2, highlighting a further reduction of 5% when analysed with CP1 and CP2 from preceding years. A further reduction was also witnessed in falls > 2 m when SP1 (−17%) was compared to SP2 (−27%). Four of the remaining categories all showed trends during SP2 towards return to pre-lockdown referral numbers. The degree of reduction (66%) seen in RTA-related referrals during SP1 decreased to a 33% reduction in SP2 when compared to CP1 and CP2, depicting more RTA-related referrals experienced in SP2 than in SP1. This was similar for falls < 2 m (−39% vs −16%), malignancy (−33% vs −2.5%), and miscellaneous (−58% vs −50%). Sporting injuries showed a complete return (−100% vs 0%) to normal values during SP2, while a notable increase in cycling-related referrals was evident (−5% vs + 63%) when compared with corresponding dates of previous years.

## Discussion

Our results highlight a large reduction in the number of referrals to our institution over the first national lockdown period for COVID-19. Reductions in the number of people presenting to hospital have been reported throughout the literature in a global setting. In England, a 25% decrease in the number of presentations to the Emergency Department was noted in the week following the initiation of a national lockdown [9]. In New Zealand, a paper published by a level 1 trauma centre reported a 43% reduction in the number of all injury admissions due to social restrictions [10], similar to the total 41% reduction we have experienced here. Reductions in referral numbers for certain MOIs can be directly or indirectly linked to the social restrictions associated with a national lockdown. For example, all sporting events in Ireland were cancelled, and thus it would be expected to see a decrease in spinal injuries related to sporting activities, as reflected in our results. Interestingly, while Morris et al. note that while overall referral numbers to their institution has decreased, the severity of injuries has remained stable [11]. However, certain reasons for referral (e.g. malignancy) may not be explained by social restrictions, and rather a decrease in hospital presentations and consultations. Reasons for this can be varied in nature, and include patient subjective fear of contracting COVID-19 in a hospital setting. It is also worth noting that failure to present or seek professional consult may be restricted by inability to access public transport during a national lockdown.

Disc disease is the most common reason for referral to the spinal unit and is usually the result of atraumatic degeneration. Therefore, it would be expected that the number of referrals for this category would have remained similar to that of previous years. However, we noted a 29% drop in referrals over the period of the lockdown. This may be due to an overall decrease in physical activity during lockdown. However, some papers have in fact reported that the general public are undertaking more physical activity than usual during the pandemic, presumably a consequence of social restrictions [12]. Furthermore, due to the typically elective nature of disc disease and logistical delays in radiology departments due to COVID-19 protection measures, cancellation or rescheduling of appointments can present a plausible reason for noted decreases in referrals for non-emergent cohorts [13]. Nevertheless, our results depict that disc-disease-related referrals have remained low post-lockdown (SP1), indicating that perhaps subjective behaviour during quarantine does have an effect on disc related referrals.

Given that the majority of these falls occur in elderly cohorts within the home, it is expected that social distancing measures would not notably affect the number injuries in this category. Easing of social restrictions and transitioning from SP1 to SP2, the number of fall referrals has remained at decreased levels compared to respective comparison periods from the preceding three years. However, the proportion of low (< 2 m) vs high (> 2 m) falls has changed slightly. During the first national lockdown (SP1), falls < 2 m saw a 39% reduction in referrals. Comparably, SP2 referral numbers were reduced by 16% when compared to CP2. Such results indicate an increase in fall (< 2 m)-related referrals as social restrictions were eased, concordant with a permitted increase of activity outside the confines of an individual’s residence or home. Similar results were witnessed with falls > 2 m. A reduction of 17% was evident when SP1 was compared to CP1, 27% when SP2 was compared with CP2. This is an interesting statistic, and may be a result of coincidence. However, it may also be a result of self-inflicted injuries due to the mental burden and social isolation associated with a national lockdown, which has been reported in certain studies [14].

In regards to other MOI categories, the reduction in referrals can be directly linked to the social restrictions implemented during the COVID-19 national lockdown. For example, sporting injuries saw a 100% reduction in referrals during the initial lockdown period (SP1), and have returned to normal levels since. RTA and cycling-related injuries saw similar patterns, potentially due to a reduction in road activity. Xiang et al. report that traffic levels and pollution have fallen significantly in major US cities [15]. Accordingly, we observed a 66% reduction in spinal injuries as a result of RTAs. As social restrictions were eased between SP1 and SP2, one would expect an increase in RTA-related referrals, conveyed by our results which report only a 37% reduction in RTA-related referrals for SP2 when compared to CP2. Interestingly, despite the reduced number of vehicles on the roads during lockdown, there was no change in the number of cycling-related injuries compared to previous years. Additionally, there was a comparative 63% increase in reported in CP2. The authors postulate that this could be due to an increase in recreational bicycle use during SP2, in addition to an increase in people attempting to avoid the use of public transport and commuting to work by bicycle.

The decrease in referrals for patients with a malignancy-related injury is a worrying trend. There is no direct causation to associate lockdown measures with the noted decrease (33%) in malignancy-related referrals. It has been documented in the literature that there have been significant disruptions in the diagnosis and treatment of cancer due to the COVID-19 pandemic, inevitably impacting on patients’ long term survival [16]. As previously mentioned, existing and prospective patients may be apprehensive to present to the hospital due to fears regarding contraction of the virus. This, accompanied by institutional logistical delays in referring units may lead to a reduction in malignancy related referrals. Such a hypothesis is somewhat validated by the results of our study, in which SP2 malignancy-related referrals have returned to near-normal (97.5%) comparative referral numbers.

## Conclusion

Spinal injury continues to occur across almost all categories, albeit at considerably reduced numbers. The social lockdown measures implemented in an attempt to flatten the transmission curve of the virus had a considerable effect on the breakdown of referrals to our institution, a national tertiary referral centre for spine pathology. In a time when healthcare resources are under strain and with fears of multiple waves of COVID-19 projected, it is important to understand referral patterns, so an effort can be made to raise awareness, minimise their occurrence, and improve overall workflow within the spinal unit. RTAs and falls remain significant mechanisms of injury during periods of social isolation. An increased projection of cycling-related referrals is plausible as increasing numbers of cyclists are evident on the roads, a trait that may remain post-lockdown measures. Particular awareness needs to be raised regarding malignancy-related spinal injuries and an effort made to dissuade people with such symptoms from avoiding presentation to hospital over periods of social restrictions.
